# A basis for comparison: sensitive authentication of stem cell derived RPE using physiological responses of intact RPE monolayers

**Published:** 2017-01-24

**Authors:** Kiyoharu J. Miyagishima, Qin Wan, Sheldon S. Miller, Kapil Bharti

**Affiliations:** 1 Section on Epithelial and Retinal Physiology and Disease, National Eye Institute, National Institutes of Health, Bethesda, MD, 20892, USA; 2 Unit on Ocular and Stem Cell Translational Research, National Eye Institute, National Institutes of Health, Bethesda, MD, 20892, USA

**Keywords:** induced pluripotent stem cells, retinal pigment epithelium, cell authentication, cellular therapy, genetic differences

## Abstract

The retinal pigment epithelium (RPE) is a monolayer of highly specialized cells that help maintain the chemical composition of its surrounding subretinal and choroidal extracellular spaces. Retinal cells (photoreceptors in particular), RPE, and choroidal endothelial cells together help ensure a homeostatically stable metabolic environment with exquisitely sensitive functional responses to light. Aging and disease of the RPE impairs its supportive functions contributing to the progressive loss of photoreceptors and vision. The prevalence of RPE associated retinal degenerations has prompted researchers to develop new therapies aimed at replacing the affected RPE with induced pluripotent stem cell (iPSC) or embryonic stem cell (ESC) derived RPE. Despite recent attempts to characterize stem cell derived RPE and to truly authenticate RPE for clinical applications, there remains a significant unmet need to explore the heterogeneity resulting from donor to donor variation as well as the variations inherent in the current processes of cell manufacture. Additionally, it remains unknown whether the starting cell type influences the resulting RPE phenotype following reprogramming and differentiation. To address these questions, we performed a comprehensive evaluation (genomic, structural, and functional) of 15 iPSC derived RPE originating from different donors and tissues and compiled a reference data set for the authentication of iPSC-derived RPE and RPE derived from other stem cell sources.

A worldwide trend towards increasing prevalence of blinding retinal degenerative diseases affecting the retinal pigment epithelium (RPE) imposes a substantial personal and economic burden on individuals and society ^[1, 2]^. The RPE is a monolayer of pigmented cells located at the back of the eye and forms the outer blood retina barrier. It maintains the volume and chemical composition of the subretinal space (SRS), and regulates nutrient and metabolite flow to and from the light sensitive photoreceptors of the retina. Several degenerative eye diseases including age-related macular degeneration (AMD) result from a disruption of structural and functional integrity of the RPE monolayer that subsequently leads to retinal degeneration.

Currently n*o* FDA-approved treatments *exist* for an advanced AMD stage called “dry” AMD where RPE cells atrophy leads to photoreceptor cell death. Multiple ongoing efforts utilize pluripotent or adult stem cells to generate healthy RPE cells as potential replacement for damaged/atrophied RPE monolayer with the goal to prevent photoreceptors loss ^[3–6]^. These efforts are founded on successful earlier studies which demonstrated that autologous RPE-choroid graft translocated from an unaffected peripheral area to the macula could lead to improved vision in AMD patients ^[7, 8]^. However, there is currently no acknowledged gold standard for what constitutes the defining characteristics of an authentically derived RPE or agreement as to how those cells can best be evaluated and selected prior to transplantation.

Pioneering work in stem cell derived RPE replacement therapy was carried out by Schwartz and colleagues at Advanced Cell Technology (ACT) - now called Astellas Institute of Regenerative Medicine. ACT initiated a clinical trial to assess the safety of a bolus injection of human embryonic stem cell (ESC)-derived RPE cells into the subretinal space of patients with Stargardt’s macular dystrophy or dry age-related macular degeneration. A preliminary report in the journal *The Lancet* revealed limited functional validation of RPE cells prior to injection in patients ^[9]^. The results in patients suggested that the injected cells were well tolerated with systemic immune suppression, but functional gains in vision remained unclear for this phase I/IIa trial ^[9, 10]^. Two other groups are following a similar approach. Cell Cure Neurosciences Ltd., based in Jerusalem is injecting a bolus of ESC-RPE in AMD patients while the Neural Stem Cell Institute, in New York is planning to inject adult RPE stem cell derived RPE cells in AMD patients. Although the transplantation approach is similar across these three potential therapies, they are markedly different in starting cells and in their manufacturing processes.

With the continued evolution in technology, other groups have altered the manufacturing process and created RPE monolayer transplants instead of cell suspension. These include the London Project to Cure Blindness, the California Project to Cure Blindness, the RIKEN, Japan initiative, and the National Eye Institute project ^[3, 11–19]^. The London and the California projects use ESC-derived RPE monolayers on plastic non-degradable scaffolds (polyester and parylene-c respectively) as the transplant support ^[11, 12, 20–22]^. In comparison, the RIKEN initiative (stopped after the first patient transplantation due to transplant manufacturing concerns) and the NEI ongoing phase I trial are planning to use autologous iPS cells. RIKEN used a collagen-based scaffold and NEI is using a biodegradable scaffold, both with the goal of having the RPE monolayer supported by their own extracellular matrix (ECM) ^[13, 23]^. Going forward, RIKEN institute and other groups (CDI/FujiFilms, Madison WI and RheinCell, Germany) have announced HLA-matched iPS cell lines for RPE-based trials for AMD and other retinal degenerative diseases. [Human leukocyte antigen (HLA) gene locus encodes for cell-surface proteins of the major histocompatibility complex (MHC). MHC class I proteins present intracellular peptides to killer T-cells. In the case of foreign cells, if T-cells do not recognize self-MHC on these antigen-presenting cells, an immune response is mounted against that cell leading to transplant rejection ^[24–27]^]. HLA-matched iPSC work is based on the hypothesis that iPSC-RPE manufactured from individuals homozygous for class I MHC HLA alleles will have a higher probability of immune acceptance in patients ^[28, 29]^. Some of the preliminary work published recently from transplantation of HLA-matched monkey iPSC-RPE showed promising results ^[30]^. The transplants were able to integrate in the back of the eye and were accompanied by minimal adaptive immune response as compared to completely allogeneic transplants that resulted in a major immune response against iPSC-RPE transplants.

Several of these ongoing and planned clinical studies are based on acceptable characterization of RPE cells including gene expression profile, ability of cells to form tight junctions, polarized cytokine secretion, and ability to phagocytose photoreceptor outer segments. It is, however, worth noting that in many cases this work was done as part of a preclinical analysis to characterize cells grown under laboratory research-grade conditions. Most of these procedures have not been validated or benchmarked for use as “release criteria” and are not validated for comparative use across multiple different studies. Several groups have urged the need for standardize practices and assays to assess adult stem cell, ESC or iPSC-derived RPE for clinical applications ^[31–33]^. Currently not known is the extent to which variability amongst donor tissues, starting stem cells, the manufacturing process, or the mode of transplantation would affect RPE cell characteristics ^[34]^. Whether several of these variables affect epigenetics of the cells while they are differentiating into the RPE lineage, thus influencing RPE cell fate and ultimately cell function is also unclear ^[35]^. Similarly it remains unknown whether the phenomenon of X-inactivation in cells of female origin ^[36]^, resulting in mosaic expression at the cellular level, would lead to variability among cells from a given female donor.

We addressed these questions by evaluating the structure, molecular, and physiological differences arising from 15 iPSC derived RPE generated from distinct tissues of several different donors. In addition to the well-established practice of verifying the typical RPE markers, gene expression profile, tight junction formation, and phagocytic ability, we employed several key functional assays (calcium imaging, electrophysiological measurements, and vectorial fluid transport) that utilize a purinergic signaling pathway with critical physiological implications to verify the structure, functional intactness and integrity of whole RPE monolayer rather than single RPE cells ^[37]^.

Our data indicate that iPS cell derived RPE exhibit several key features of primary human RPE cells: demonstrating typical RPE morphology and structure, expressing RPE signature genes, microRNAs and protein markers ^[37]^. However, we found that the functional analysis of the ATP-mediated purinergic signaling pathway provides the most sensitive readout of RPE authenticity highlighting variation among RPE derived from different tissues and donors ^[37]^ (Fig. 1). Most importantly, we were able to identify several iPSC derived RPE samples that possessed RPE-like qualities (similar RPE gene expression, key RPE protein markers and phagocytic ability) but failed to meet the more stringent functional requirements deemed critical for authentic RPE. These stringent functional assays are vital to ensure that any stem cell derived RPE are properly polarized and capable of performing the RPE’s complex diverse functions to provide therapeutic benefit upon transplantation into the subretinal space ^[37]^. Our paper draws attention to the benefit of systematically assessing the function of the intact RPE monolayer as an improved release criterion for RPE derived from iPS cells or other stem cells for clinical application.

In our recent report we attempt to define the major sources of variability and establish the acceptable limits of variability (epigenetic, and technical/manufacturing). We demonstrate that the basis for functional variation stems from manufacturing/technical heterogeneity, which exceeds epigenetic influences of the starting tissue. Additionally, we found that clonal variability among iPSC-RPE derived from female donors is relatively low suggesting that X-chromosome inactivation does not strongly influence RPE function ^[37]^.

In summary, we have compiled a reference data set that encompasses genomic, structural, and functional variation and can be used to compare and authenticate any stem cell derived-RPE derived for therapeutic or research purposes. This data set will allow groups to select one release criterion that can be used to compare clinical RPE product across different trials. Our work provides a range of values expected for most RPE functions within which RPE cells can be deemed authentic. We believe that this data will have significant practical benefit and suggest helpful guidelines as a basis for further improvements to the scientific community and will help translate RPE into commercially successful clinical applications.

## Figures and Tables

**Figure 1:**
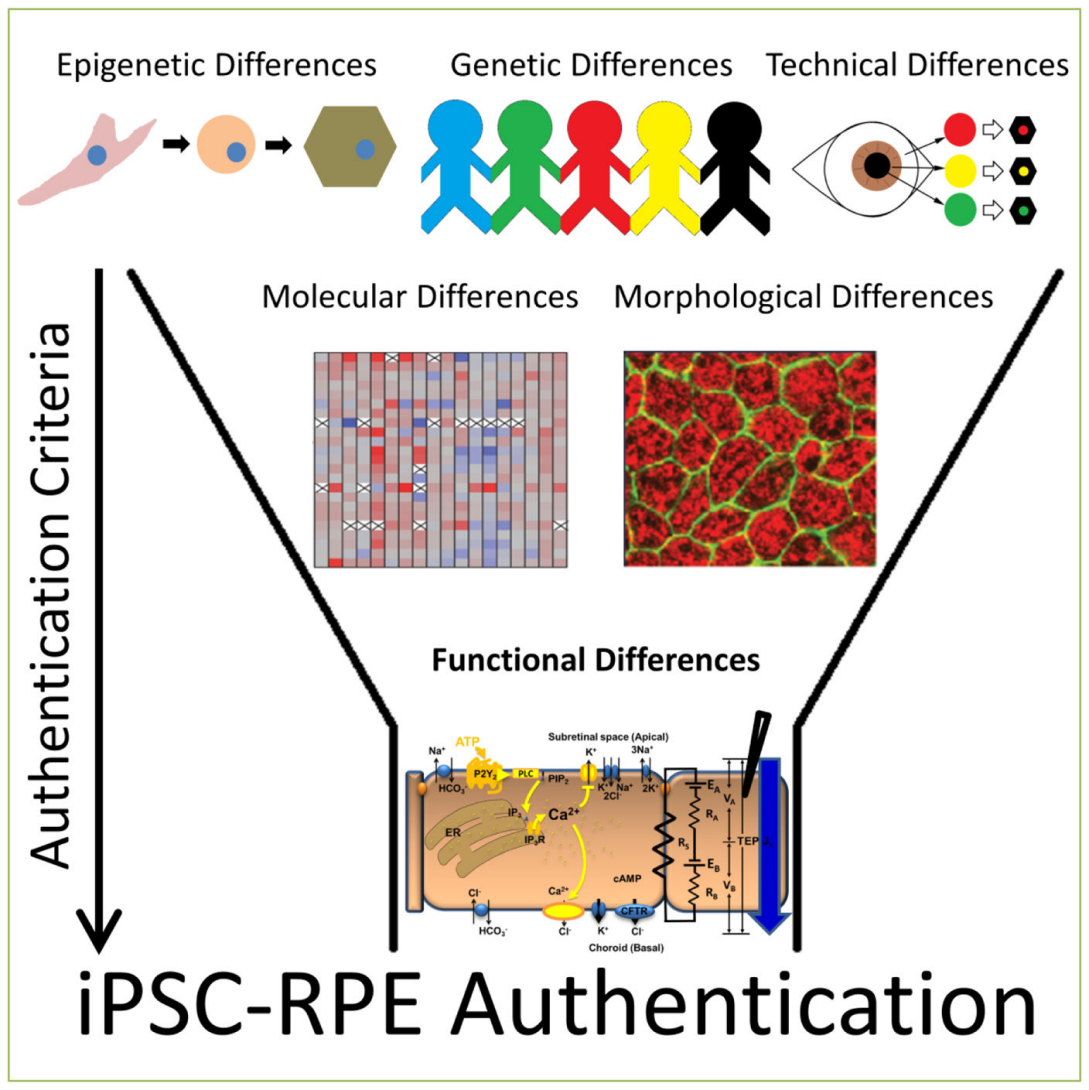
iPSC-RPE Authentication The inherent variability in populations of iPSCs can influence the formation and organization of functional iPSC-derived RPE monolayers. (Top) Sources of variability include -predominantly genetic differences between donors, but also epigenetic and clonal heterogeneity (technical differences). (Middle) This variability makes it difficult to distinguish authentic RPE solely at the molecular or morphological level. (Bottom) This paper illustrates how an ATP-dependent signaling pathway that drives critical aspects of RPE function can be used to more globally assess the functional characteristics of the entire RPE monolayer and authenticate fully differentiated RPE cells.

## Abbreviations

iPSCs: induced pluripotent stem cells
RPE: retinal pigment epithelium
ESC: embryonic stem cell
SRS: subretinal space
AMD: age-related macular degeneration
ACT: Advanced Cell Technology
ECM: extracellular matrix
HLA: Human leukocyte antigen
MHC: histocompatibility complex
